# In-vivo properties and functional subtypes of gonadotropin-releasing hormone neurons

**DOI:** 10.1016/j.isci.2025.112513

**Published:** 2025-04-22

**Authors:** Yali Liu, Xi Shen, Yuqi Zeng, Yunhan Nie, Jiamin Xu, Allan E. Herbison, Yanping Kuang, Li Wang

**Affiliations:** 1 Department of Assisted Reproduction, Shanghai Ninth People’s Hospital, Shanghai JiaoTong University School of Medicine, Shanghai 200003, P.R. China; 2 Department of Physiology, Development and Neuroscience, University of Cambridge, CB2 3EG Cambridge, UK; 3 Key Laboratory of Brain Functional Genomics, East China Normal University, Shanghai 200062, P.R. China

**Keywords:** Natural sciences, Biological sciences, Neuroscience, Cellular neuroscience

## Abstract

Although the gonadotropin-releasing hormone (GnRH) neurons play a pivotal role in mammalian fertility, their activity patterns and functional classification during ovulation and across reproductive cycles still remain elusive. By integrating *in vivo* electrophysiological recording with chemogenetic technology, we successfully identified GnRH neurons in freely moving mice. Recordings obtained revealed that the baseline firing rates of rostral preoptic area (rPOA) GnRH neurons were significantly higher during proestrus compared to the metestrous and estrous stages of the cycle. Continuous 20-h recordings showed surge-like activity in a subpopulation of GnRH neurons (∼47%) during the proestrous stage, characterized by diverse firing patterns, while other GnRH neurons exhibited basal rhythmic firing patterns. The similar waveform characteristics of GnRH neurons facilitate their identification for subsequent *in vivo* electrophysiology studies. This study represents a significant milestone in functionally classifying GnRH neurons *in vivo* and holds great importance for investigating neuroendocrine regulation of female reproduction.

## Highlights

• GnRH neurons were successfully identified by *in-vivo* electrophysiology
• The baseline firing rates of GnRH neurons were highest during proestrus
• Half of the GnRH neurons were involved in the LH surge, with diverse activity patterns
• Other GnRH neurons were either quiescent or exhibited basal rhythmic activity

## Graphical abstract

**Figure:**
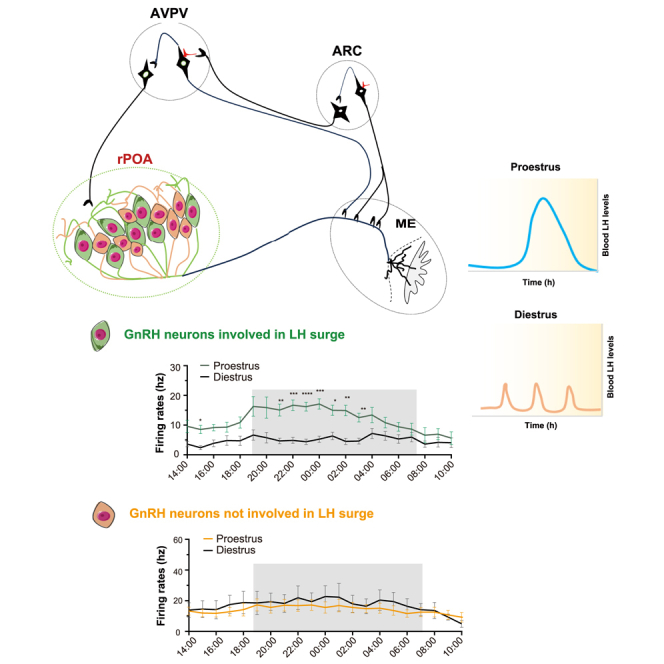

## Introduction

The gonadotropin-releasing hormone (GnRH) neurons serve as the ultimate output cells of the reproductive neuronal network in the hypothalamus and play a pivotal role in female fertility.^1^^,^^2^ The GnRH neuron cell bodies are widely scattered throughout the rostral pre-optic area (rPOA) and basal hypothalamus but send projections to the median eminence where they release GnRH into the portal system.^3^ Evidence suggests that the cell bodies and proximal dendrites of GnRH neurons play a crucial role in generating GnRH surges, while neural processing at their distal projections near the median eminence drive pulsatile GnRH secretion.^4^^,^^5^ The generation of GnRH surges and pulses is essential for female reproductive cyclical activity and spontaneous ovulation.^6^^,^^7^ During the follicular phase, GnRH neurons generate approximately one pulse per hour, which significantly slows to one pulse every 2 to 3 h during the luteal phase.^8^^,^^9^ In females, a rise in estradiol secretion from maturing follicles during the mid-cycle provides an estrogen positive feedback signal essential for generating the preovulatory surge secretion of GnRH.^10^^,^^11^ Despite the role of GnRH neurons as pivotal regulators of female reproduction, much remains unknown about their *in vivo* activity and functional classification across the estrous or menstrual cycle.

In the past few decades, diverse methodologies have been utilized to investigate the activity and function of GnRH neurons. However, many of the existing approaches still rely on indirect endpoint measures. For instance, assessing acute stimulus-induced changes in the immediate-early gene c-Fos as an indicator of electrical activation has demonstrated that GnRH neurons are indeed activated concurrently with the occurrence of the GnRH surge.^12^^,^^13^ Electrophysiological studies in acute brain slices have also recorded the electrical activities and properties of individual GnRH neurons.^14^ These investigations have revealed a diverse range of firing patterns exhibited by GnRH neurons, including bursting, continuous activity, and silence.^15^^,^^16^ It is important to note that afferent inputs to GnRH neurons are severed during brain slice preparation,^17^ thus resulting in the firing patterns observed under *in vitro* conditions not aligning well with predicted pulse or surge patterns of GnRH secretion.^14^^,^^18^

The utilization of promoter-driven genetic techniques has greatly facilitated investigations into the function and properties of GnRH neurons.^19^ However, the scattered distribution of GnRH cell bodies across the hypothalamus and the distal site of GnRH release presents a formidable obstacle to studying the *in vivo* activity and function of individual GnRH neurons.^3^ To date, only a single study has reported on the electrophysiological properties of anterior hypothalamic area (AHA) GnRH neurons *in vivo*, but this was performed in anesthetized animals.^20^

Advancements in multielectrode recording techniques^21^^,^^22^ coupled with genetic approaches have now allowed us to make simultaneous recordings of individual GnRH neurons in intact, freely moving mice. We utilized an array of 32 nickel-chromium electrode wires to maximize anatomical coverage of the rPOA and shaped the coronal plane of the microelectrode into an inverted Y shape to align with the distribution pattern of GnRH neurons. This has allowed us to identify GnRH neurons *in vivo* and track their physiological activities during proestrus and throughout different estrous cycles. This research demonstrates both fundamental insights into the *in vivo* activity patterns of GnRH neurons and provides a functional classification of this key neuronal cell type.

## Results

### Characterization of GnRH-Cre::hM4D(Gi)/hM3D(Gq) mice

**Figure 1:**
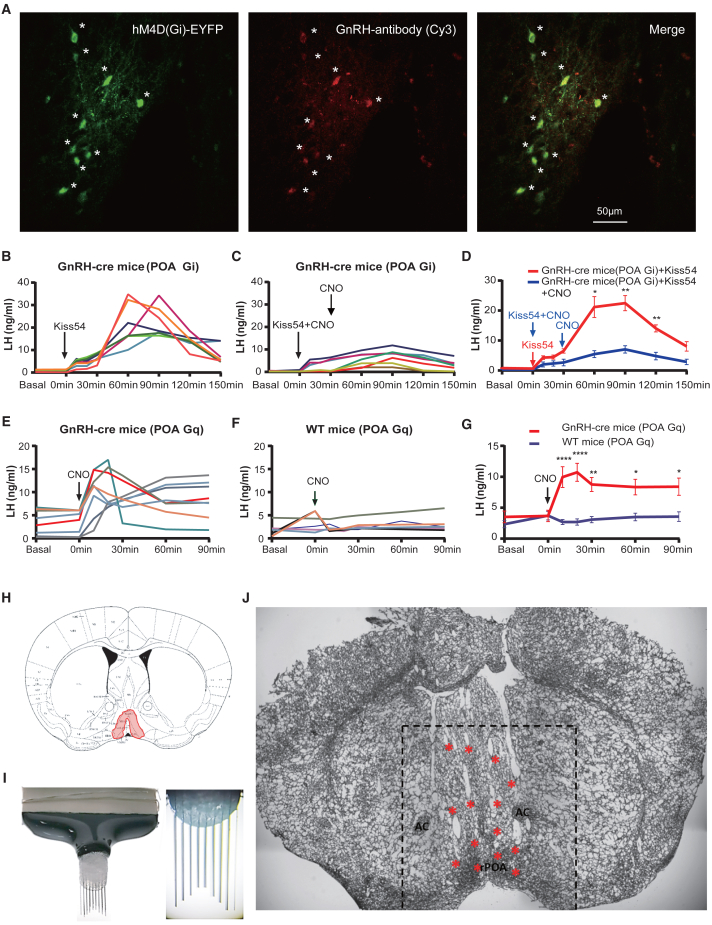
The GnRH-cre dependent chemogenetic AAVs were validated for their expression and function, followed by implantation using a custom 32-channel electrode (A) The photomicrographs depict the presence of GnRH immunoreactivity (in red), hM4D(Gi)- EYFP expression (in green), and their overlay in GnRH-Cre mice that received bilateral injections of Cre-dependent DIO- hM4D(Gi)- EYFP AAVs into the rostral preoptic area (rPOA). Asterisks indicate GnRH neurons expressing hM4D(Gi)-EYFP. Scale bar, 50 μm. (B and C) LH profiles were obtained for all GnRH::DIO-hM4D(Gi)-EYFP mice administered with Kiss54 alone or in combination with CNO (*n* = 7). (D) The LH levels (mean ± SEM) in GnRH::DIO-hM4D(Gi)-EYFP mice were compared when administered with Kiss54 alone or in combination with CNO. ∗*p* < 0.05; ∗∗*p* < 0.01, two-way repeated measures ANOVA with Holm-Sidak test. (E and F) The LH profiles were obtained for the GnRH::DIO-hM3D(Gq)- EYFP mice (E, *n* = 8) or WT::DIO-hM3D(Gq)- EYFP mice (F, *n* = 7) administered with CNO. (G) The LH levels (mean ± SEM) were compared between GnRH::DIO-hM3D(Gq)-EYFP and WT::DIO-hM3D(Gq)-EYFP groups administered with CNO. ∗*p* < 0.05, ∗∗*p* < 0.01, ∗∗∗∗*p* < 0.0001, two-way repeated measures ANOVA with Holm-Sidak test. (H) The schematic illustrates the anatomical boundaries of the rostral preoptic area (rPOA). (I) The customized 32-fixed channel electrode with an inverted Y shape designed for the rPOA brain region. (J) rPOA brain map displays the locations where 32 channel electrodes are embedded. Asterisks indicate traces of electrode filament entry into brain slices.

Following the injection of AAV 2/9 Cre-dependent hSyn-DIO-hM4D(Gi)-EYFP or EF1a-DIO-hM3D(Gq)-EYFP into the rPOA of Gnrh1-Cre^+/−^ mice, dual label immunohistochemistry revealed that approximately 80% of rPOA GnRH neurons expressed hM4D(Gi) (Figure 1A) or hM3D(Gq) EYFP (Figure S1A).

The i.p. administration of Kiss-54 to GnRH:hM4D(Gi) mice resulted in a marked elevation in plasma luteinizing hormone (LH) levels that peaked at 60–90 min and lasted for approximately 120 min (Figure 1B, *n* = 7). The co-administration of Kiss-54 with CNO 30 min later resulted in significantly lower LH levels at the 60 min (*p* < 0.05), 90 min (*p* < 0.01) and 120 min time points (*p* < 0.01, two-way repeated measures ANOVA with Holm-Sidak test) (Figures 1C and 1D, *n* = 7).

The i.p. administration of CNO to GnRH:hM3D(Gq) mice resulted in an abrupt increase in LH secretion within 10 min that remained elevated for over 90 min (Figure 1E, *n* = 8). No change in LH levels was detected in control hM3D(Gq) mice given CNO (Figure 1F, *n* = 7). Elevated LH levels were significantly higher at 10min, 20min, 30min, 60min and 90min compared to those of control mice (∗*p* < 0.05, ∗∗*p* < 0.01, ∗∗∗∗*p* < 0.0001, two-way repeated measures ANOVA with Holm-Sidak test) (Figure 1G).

### Dual identification criteria for identifying GnRH neurons in GnRH-cre^+/−^ mice

A custom-designed 32-channel electrode with an inverted Y shape (Figure 1I) was implanted into the rPOA brain region (Figure 1H). A representative example of electrode locations is depicted in the coronal plane in Figure 1J.

We used dual criteria for identifying GnRH neurons in GnRH:hM4D(Gi) and GnRH:hM3D(Gq) mice. Given the selective expression of DREADDs in GnRH neurons of our mouse models, we used the response of individual neurons to CNO as one criterion. As the expression of kiss1r is also restricted to GnRH neuron cell bodies in the rPOA,^23^ we used the response of a cell to kisspeptin as a second criterion. For these experiments, we used Kiss-10 due to its relatively short half-life that generates a robust but more refined stimulation of LH secretion (Figures S1B and S1C). While neither test alone ensures the cell recorded is directly activated by CNO or kisspeptin, we reasoned that cells that respond to both tests had a very high likelihood of being a GnRH neuron. We first tested the response of individual neurons with Kiss-10 and then probed with CNO.

**Figure 2:**
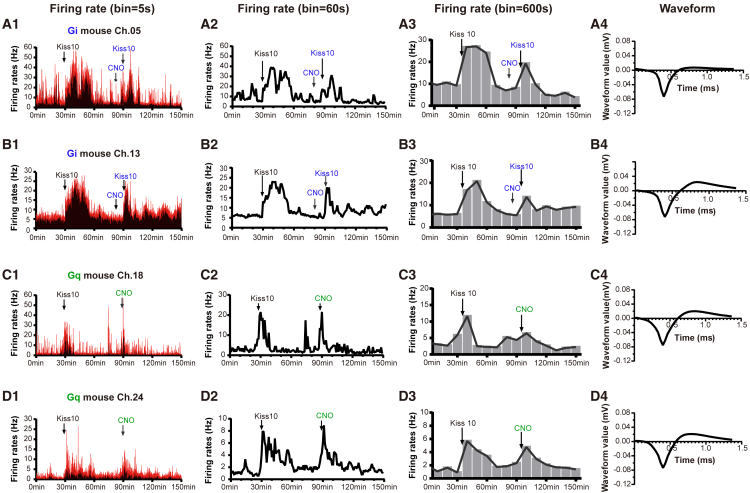
Examples of GnRH neurons identified in the GnRH::DIO-hM4D(Gi) and GnRH::DIO-hM3D(Gq) mice through Kiss 10 and CNO injection (A1–B4) The firing rates (A1, B1, A2, B2, A3, and B3) and waveforms (A4 and B4) of two representative GnRH neurons were determined by administering Kiss10 and Kiss10+CNO in GnRH::DIO-hM4D(Gi) Mice. The identification criterion for GnRH neurons: The excitatory increase in firing induced by Kiss-10 should be reduced by >40% by the prior administration of CNO. The excitatory rate induced by Kiss-10 was calculated as the disparity between firing rates during the first 10 min after Kiss-10 injection and baseline firing rates over a 30-min period, divided by the baseline firing rates. Similarly, the excitatory rate induced by both Kiss-10 and CNO was determined as the discrepancy between firing rates during a 30-min period following the co-injection of Kiss-10 with CNO and baseline firing rates over a 30-min period, divided by the baseline firing rates. (C1–D4) The firing rates (C1, D1, C2, D2, C3, and D3) and waveforms (C4 and D4) of two representative GnRH neurons were determined by injecting Kiss10 and CNO in GnRH::DIO-hM3D(Gq)- EYFP Mice. The identification criterion for GnRH neurons was that both the excitatory rate induced by Kiss-10 alone and the excitatory rate induced by CNO should exceed 40% of the basal firing rate. The excitatory rate induced by Kiss-10 was calculated as the firing rates during the first 10 min after Kiss10 injection minus the baseline firing rates over a period of 30 min, divided by the baseline firing rates over 30 min. Similarly, the excitatory rate induced by CNO was calculated as the firing rates during a 30-min period following CNO injection minus the baseline firing rates over a period of 30 min, divided by the baseline firing rates over 30 min.

For GnRH:hM4D(Gi) mice, stable recordings of rPOA neurons were made in multiple channels simultaneously, and Kiss-10 was administered. If cells were identified to increase their firing rates, another Kiss-10 injection was given 60 min later but preceded by 10 min with an i.p. injection of CNO (Figures 2A and 2B). In the representative examples shown in Figure 2, the neuron recorded from channel 5 (Ch.05) was robustly activated by kisspeptin (174% increase in firing), and this was greatly attenuated by pre-treatment with CNO (16% increase) (Figure 2A). Similarly, the firing rate of the neuron recorded in Ch.13 was enhanced by Kiss-10 (180%), and this was reduced (72%) when CNO was given before the second Kiss-10 treatment (Figure 2B). As a conservative approach, we defined GnRH neurons as recorded units in which Kiss-10 activated firing and pre-treatment with CNO suppressed this activation by >40%. The waveforms of the Ch.05 and Ch.13 units are depicted in Figures 2A4 and 2B4, respectively. The same GnRH neuron identified by Kiss-10 and CNO (Figures S2B1–S2B4) could also be identified using Kiss-54 and CNO (Figure S2A1–S2A4). Intraperitoneal injection of saline did not induce significant alterations in the firing rates of GnRH neurons in either the GnRH:hM4D(Gi) or GnRH:hM3D(Gq) mouse lines (Figures S2C1–S2D4).

For GnRH:hM3D(Gq) mice, stable recordings were obtained, and a Kiss-10 test was applied as above. If units responded, the mouse was then given i.p. CNO 60 min later (Figures 2C and 2D). In Figure 2C, the firing rate of the unit recorded in Ch.18 was significantly increased by Kiss-10 (200% increase over basal firing) and also excited by CNO administration (65% increase) (Figures 2C1–2C3). Similarly, the excitatory rate of the Ch.24 unit showed a significant increase upon Kiss-10 treatment (396%) and was also activated by CNO administration (293%) (Figures 2D1–2D3). For this approach, a unit was considered a GnRH neuron if both Kiss-10 and CNO increased firing by >40% of basal rates. The waveforms of Ch.05 and Ch.20 are depicted in Figures 2C4 and 2D4, respectively.

Overall, 17% of recorded rPOA units (*N* = 9 in 54 recorded units) were identified as being GnRH neurons using the GnRH:hM4D(Gi) mouse model, and 14% of rPOA units (*N* = 6 in 43 recorded units) using the GnRH:hM3D(Gq) model. Identified GnRH neurons recorded from both mouse models were combined to generate the following data.

### Activity patterns of GnRH neurons across the estrous cycles

**Figure 3:**
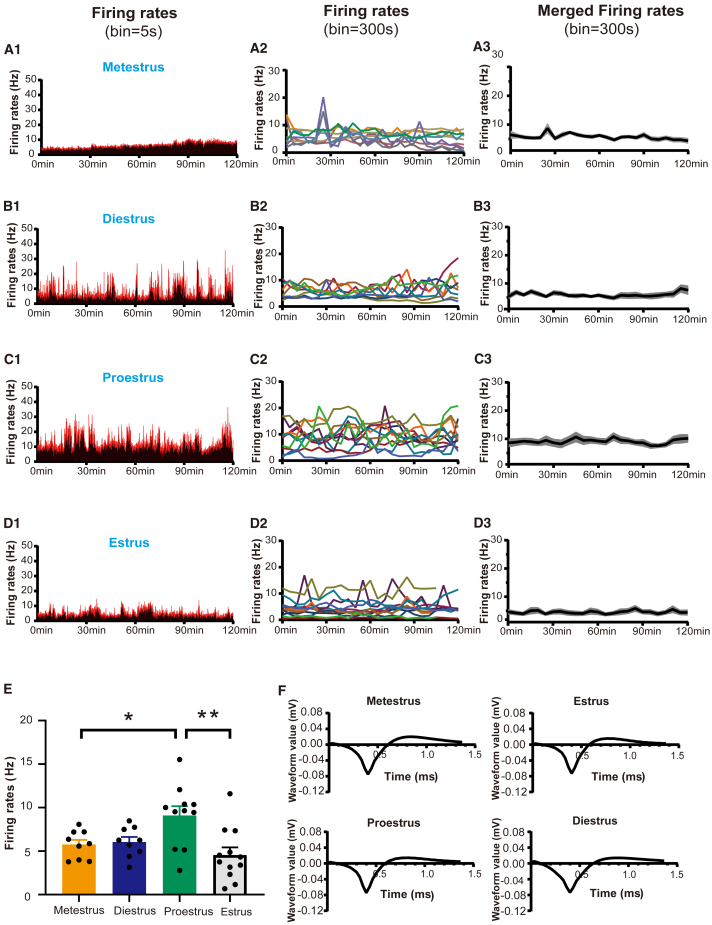
The Profiles of basal firing rates in the morning across different estrous cycles (A1–D3) Representative example of the basal firing rates (bin = 5s) across different stages of the estrous cycle recorded from 10:00 to 12:00 (A1, B1, C1, and D1). The entire basal firing rate profile (bin = 300s) for GnRH neurons during metestrus is shown in A2 (*n* = 9 recordings from 6 neurons in 5 mice), diestrus in B2 (*n* = 9 recordings from 7 neurons in 6 mice), proestrus in C2 (*n* = 11 recordings from 7 neurons in 6 mice), and estrus in D2 (*n* = 12 recordings from 7 neurons in 6 mice), along with their respective mean levels depicted as mean ± SEM firing rates at bin size of 300s shown as A3, B3, C3, and D3. (E) Mean ± SEM basal firing rates of GnRH neurons during metestrus, diestrus, proestrus, and estrus are presented. Statistical significance was determined using one-way ANOVA followed by Tukey’s post hoc test (∗*p* < 0.05, ∗∗*p* < 0.01). (F) Mean ± SEM waveforms corresponding to different stages of the estrous cycle.

In total, *in vivo* electrophysiological recordings were made from 6 to 7 GnRH neurons in 5–6 female mice in the mornings of metestrus, diestrus, proestrus, and estrus. After identifying GnRH neurons in freely behaving mice, their basal firing rates were recorded for 2-h periods between 10 a.m. and 12 a.m. on different days of the estrous cycle as determined by vaginal cytology. The firing rate profiles of GnRH neurons exhibited a variable baseline pattern in metestrus (*n* = 9 recordings from 6 neurons in 5 mice) (Figures 3A1 and 3A2), diestrus (*n* = 9 recordings from 7 neurons in 6 mice) (Figures 3B1 and 3B2), proestrus (*n* = 11 recordings from 7 neurons in 6 mice) (Figures 3C1 and 3C2), and estrus (*n* = 12 recordings from 7 neurons in 6 mice) (Figures 3D1 and 3D2). The merged firing rates with bins set at intervals of every 300 s were analyzed for metestrus, diestrus, proestrus, and estrus as shown in Figures 3A3, 3B3, 3C3, and 3D3.

The average basal firing rates during the four stages of the estrous cycle are presented in Figure 3E. The highest rate was observed during proestrus (9.10 ± 1.07 Hz), which was significantly higher than that during estrus (4.55 ± 0.88 Hz) (*p* < 0.01) and metestrus (5.76 ± 0.54 Hz) (*p* < 0.05, parametric one-way ANOVA with post hoc Tukey’s multiple comparisons test). However, there was no significant difference with diestrus (6.05 ± 0.57 Hz). The waveforms of individual neurons at different stages of the estrous cycle exhibited no discernible variations (Figure 3F). The basal activity of GnRH neurons across four different estrous cycles at the same GnRH neuron (Figure S3) shows similar activity patterns to those observed in the GnRH population depicted in Figure 3.

### Identification of a GnRH neuron sub-population exhibiting surge-like activity during proestrus

In total, extended *in vivo* electrophysiological recordings were made from 15 GnRH neurons in 6 female mice over a 20-h across proestrus into estrus. Of these, 7 exhibited changes in firing rate over the proestrus-estrus period but not from diestrus-proestrus morning, while the 8 remaining neurons exhibited unchanging patterns of activity on both days of the cycle.

**Figure 4:**
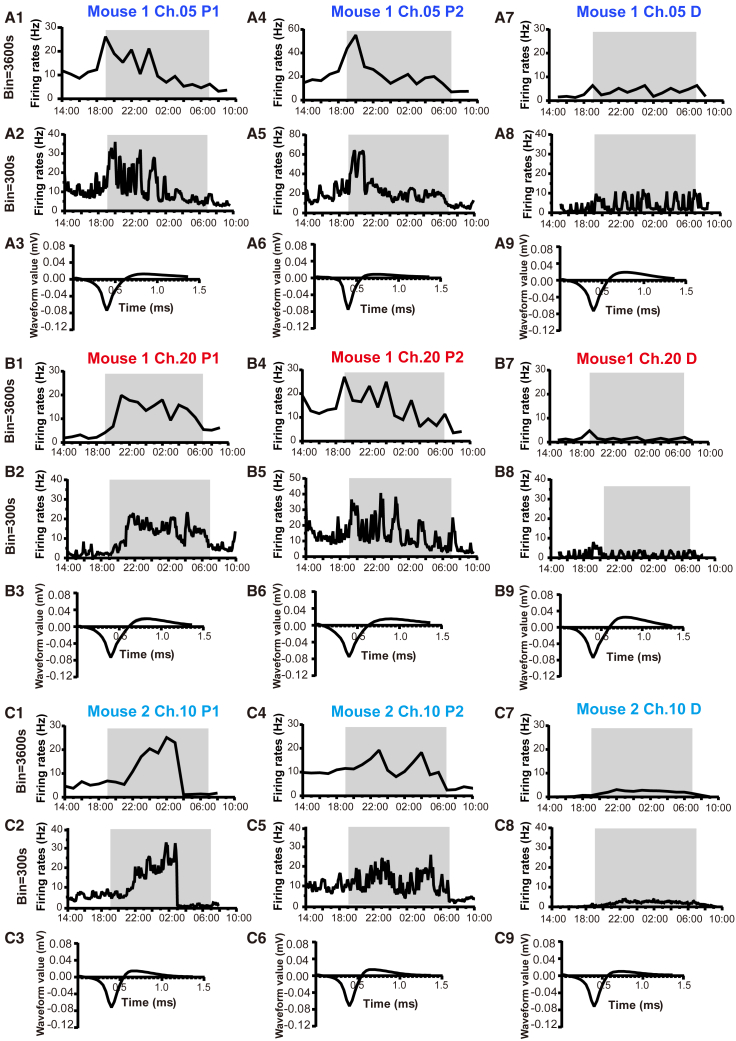
*In-vivo* electrophysiological recordings of three GnRH neurons across two proestrus cycles (A1–A9) The firing rates and waveforms (A3, A6, and A9) of a single GnRH neuron were recorded in GnRH::DIO-hM4D(Gi)-EYFP Mice #1 Ch.05) during 20-h *in-vivo* electrophysiological recordings encompassing two proestrus cycles (A1–A3: the first proestrus; A4-A6: the second proestrus) and one diestrus cycle (A7–A9). Changes in firing rate were analyzed at intervals of 300s (A1, A4, and A7). (B1–B9) The firing rates and waveforms (B3, B6, and B9) of the second GnRH neuron were recorded in GnRH::DIO-hM4D(Gi)-EYFP Mice (#1 Ch.20) during 20-h *in-vivo* electrophysiological recordings encompassing two proestrus cycles (B1–B3: the first proestrus; B4–B6: the second proestrus) and one diestrus cycle (B7–B9). Changes in firing rate were analyzed at intervals 300s (B1, B4, and B7). (C1–C9) The firing rates and waveforms (C3, C6, and C9) of the third GnRH neuron were recorded in GnRH::DIO-hM4D(Gi)-EYFP Mice (#2 Ch.10) during 20-h *in-vivo* electrophysiological recordings encompassing two proestrus cycles (C1–C3: the first proestrus; C4–C6: the second proestrus) and one diestrus cycle (C7–C9). Changes in firing rate were analyzed at intervals 300s (C1, C4, and C7).

**Figure 5:**
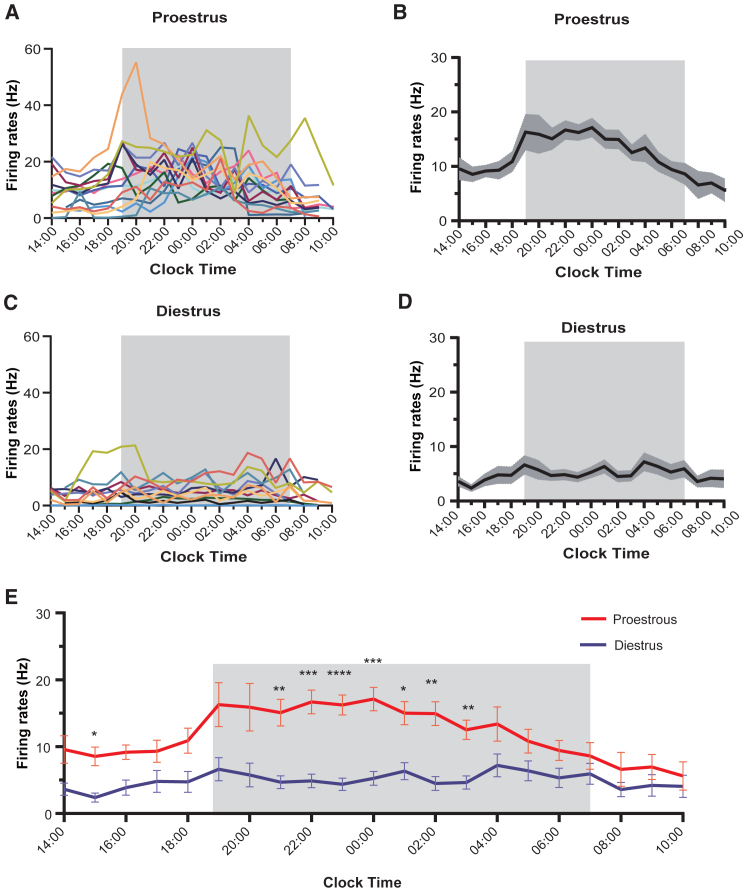
The *in-vivo* electrophysiological activities of some GnRH neurons exhibit surge-like patterns during proestrous (A–E) The firing rates (bin = 3600s) of this specific type of GnRH neuron during proestrous (A, *n* = 14 recordings from 7 neurons in 6 mice) and the related mean (±SEM) firing rate levels (B). The firing rates (bin = 3600s) of this specific type of GnRH neuron during diestrus (C, *n* = 11 recordings from 7 neurons in 6 mice) were assessed overall and the related mean (±SEM) firing rate levels (D), as well as a comparison of mean (±SEM) firing rate levels between the two groups (E). ∗*p* < 0.05, ∗∗*p* < 0.01, ∗∗∗*p* < 0.001, ∗∗∗∗*p* < 0.0001, two-way repeated measures ANOVA with Holm-Sidak test.

The firing rates of three different GnRH neurons recorded from two mice (#1 Ch.05, #1 Ch.20, #2 Ch.10) from 14:00 on proestrus to 10:00 on estrus on two different estrous cycle stages and on diestrus-metestrus are shown in Figures 4A–4C. Considering the first proestrus recordings, all units exhibited a robust increase in activity centered around the time of lights-off with different temporal patterns of firing with oscillatory as well as relatively consistent increases in firing rate over several hours (Figures 4A1, A2, B1, B2, C1, and C2). By ensuring that the waveforms of identified units remained the same (Figures 4A3, A6, A9, B3, B6, B9, C3, C6, and C9) it was possible to record the activity of the same GnRH neuron on different estrous cycle. One GnRH neuron (Ch.05) exhibited an identical increase in firing rate at lights-off but then stopped firing 2h later (Figure 4A5). Another (Ch.20) continued to exhibit a prolonged pattern of increased activity but this was more oscillatory than before (Figure 4B5). While another GnRH neuron (Ch.10) exhibited relatively little dynamic change in activity during the second proestrus (Figure 4C5). All three neurons displayed a low level of relatively consistent firing across diestrus (Figure 4A7, A8, B7, B8, C7, and C8). The firing rates of all “surge-like” GnRH neurons recorded over the 20-h proestrus-estrus (*n* = 14 recordings from 7 neurons in 6 mice) and diestrus-proestrus (*n* = 11 recordings from 7 neurons in 6 mice) periods are depicted in Figure 5. On proestrus, a gradual increase in baseline activity was observed starting at approximately 15:00 (4.0 h before "lights-off"), reaching its peak at midnight, and returning to baseline by 8:00 a.m. The mean ± SEM firing rates of GnRH neurons started at a baseline frequency of 8.54 ± 1.4 Hz, peaked at the highest rate of 17.12 ± 1.76 Hz, and returned to a baseline level of 6.59 + 2.55 Hz by morning (Figure 5B). No similar fluctuations in firing were observed in the diestrous-proestrous morning recordings (Figure 5D). The firing rates during proestrus were significantly higher from 21:00 to 3:00 a.m. compared to those during diestrus (∗*p* < 0.05, ∗∗*p* < 0.01, ∗∗∗*p* < 0.001, ∗∗∗∗*p* < 0.0001, two-way repeated measures ANOVA with Holm-Sidak test) (Figure 5E).

**Figure 6:**
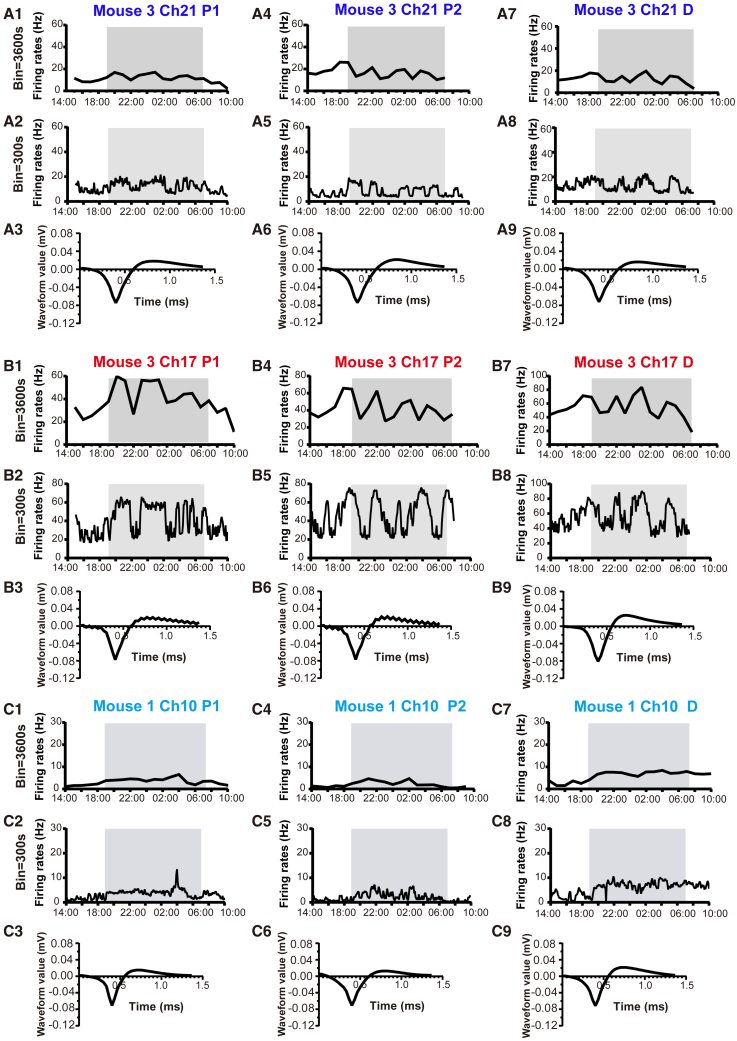
The *in-vivo* electrophysiological activities of GnRH neurons exhibit “quiescence” or rhythmic firing patterns throughout both proestrus and diestrus (A1–A9) A single GnRH neuron, recorded in GnRH::DIO- hM3D(Gq)- EYFP Mice (#3 Ch21) during 20-h *in-vivo* electrophysiological recordings, exhibits consistent basal electrophysiological activity and waveforms (A3, A6, and A9) throughout two proestrus cycles (A1–A3: the first proestrus; A4–A6: the second proestrus) and one diestrus cycle (A7–A9). Changes in firing rate were analyzed at intervals of 3600s (A1, A4, and A7) and 300s (A2, A5, and A8). (B1–B9) The second GnRH neuron, recorded in GnRH::DIO- hM3D(Gq)- EYFP Mice (#3 Ch17)during 20-h *in-vivo* electrophysiological recordings, demonstrates consistent basal electrophysiological activity and waveforms (B3, B6, and B9) across two proestrus cycles (B1–B3: the first proestrus; B4–B6: the second proestrus) and one diestrus cycle (B7–B9). Changes in firing rate were analyzed at intervals of 3600s (B1, B4, and B7) and 300s (B2, B5, and B8). (C1–C9) The third GnRH neuron was recorded from GnRH::DIO-hM4D(Gi)-EYFP Mice (#1 Ch10) during a continuous 20-h period of *in-vivo* electrophysiological recordings. It exhibited consistent basal electrophysiological activity and waveforms (C3, C6, and C9) throughout two proestrus cycles (C1–C3: the first proestrus; C4-C6: the second proestrus), as well as one diestrous cycle (C7–C9). Changes in firing rate were analyzed at intervals of 3600s (C1, C4, and C7), as well as every 300 s thereafter.

The firing rates of three GnRH neurons did not change substantially at any time across the 20-h proestrus-estrus or diestrus-proestrus recording periods (Figure 6). These cells showed a range of different firing patterns that were, nevertheless, similar on the different days of the cycle. Two exhibited a clearly oscillatory pattern of activity with periodic activity lasting hours (Figures 6A and 6B) while the other was relatively stable (Figure 6C).

### Waveform characteristics of recorded GnRH neurons

**Figure 7:**
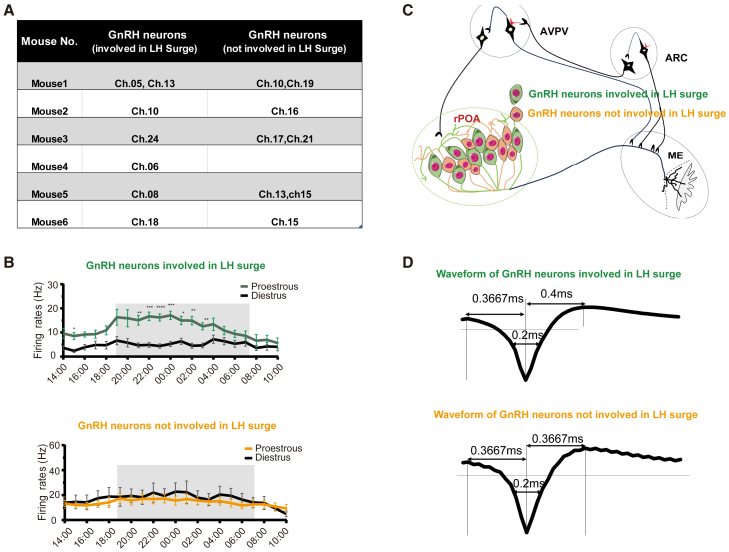
Summary of recorded GnRH neurons (A) The recording of 15 GnRH neurons in 6 mice was documented. (B) A comparative analysis of average firing rates of different subtype GnRH neurons during proestrus and diestrus. ∗*p* < 0.05, ∗∗*p* < 0.01, ∗∗∗*p* < 0.001, ∗∗∗∗*p* < 0.0001, two-way repeated measures ANOVA with Holm-Sidak test. (C) The pattern diagram illustrates the two distinct types of GnRH neurons in rPOA. (D) The waveform characteristics of the two types of GnRH neurons.

We recorded *in vivo* electrophysiological activity of 15 GnRH neurons in 6 mice. Among these neurons, 7 were responsible for triggering the LH surge during proestrus, while the remaining 8 remained inactive for a period of 20 h (Figure 7A). One sub-population of GnRH neurons exhibited surge-like activity during proestrus (∼47%), while another sub-population of GnRH neurons remained either "quiet" or exhibited a basal rhythmic firing pattern during proestrus and diestrus (∼53%, Figures 7A and 7B). The pattern diagram in Figure 7C illustrates the two sub-population of GnRH neurons in the rPOA.

The waveforms of GnRH neurons exhibit characteristic features irrespective of whether they are involved in the surge (Figure 7D). The duration between the left peak and minimum was found to be 0.3667ms for both types, while the duration between the minimum and right peak was 0.4ms for “surge” GnRH neurons and 0.3667ms for “non-surge” GnRH neurons. Additionally, the half-width between two points in the waveform that have an amplitude equal to half of the maximum peak to-peak amplitude was measured as 0.2ms for both type of GnRH neurons.^24^^,^^25^^,^^26^ Due to the lower proportion of metestrus (∼0.5 days) and estrus (∼1 day) within the entire estrous cycle (∼4–5 days),^27^ we traced and presented the overnight recording data of the same GnRH neurons only for the proestrus and diestrus stages to highlight the differences, but did not show the limited overnight recordings during other stage transitions. However, several overnight recordings of some GnRH neurons without subtypes across the transition from estrus to metestrus (*n* = 5 recordings from 3 neurons in 2 mice), and from metestrus to diestrus (*n* = 5 recordings from 4 neurons in 2 mice) are shown in Figure S4.

## Discussion

We provide here the initial direct recordings of rPOA GnRH neuron activity *in vivo*. Our observations demonstrate a significant increase in baseline firing rates of rPOA GnRH neurons during proestrus compared to metestrus and estrus. Prolonged 20-h *in vivo* recordings throughout proestrus into estrus revealed surge-like firing rates from specific subpopulations of GnRH neurons that would sufficiently account for the surge patterns of LH secretion in females. Not all rPOA GnRH neurons are necessarily active during the LH surge with approximately 47% GnRH neurons exhibiting surge-like activity during this phase according to our study findings. The remaining GnRH neurons displayed consistent or highly variable oscillatory firing patterns. The waveforms produced by these two subpopulations of GnRH neurons appear quite similar, facilitating their identification in subsequent *in vivo* electrophysiology studies.

We used dual identification criteria for identifying GnRH neurons in the GnRH:hM4D(Gi) and GnRH:hM3D(Gq) mice: The intraperitoneal administration of CNO in GnRH:hM4D(Gi) mice would be expected to lead to the activation of inward-rectifying potassium channels that suppress electrical excitability and inhibit GnRH neuron activity.^28^^,^^29^ In contrast, CNO would be expected to activate the phospholipase C signaling cascade in GnRH neurons in GnRH:hM3D(Gq) mice leading to heightened activity.^28^^,^^29^ Optogenetics is more direct and responsive compared to chemogenetics; however, in our preliminary experiments, no GnRH neurons were successfully recorded using optogenetic approaches. The scattered distribution of GnRH cell bodies across the hypothalamus^3^ poses a significant challenge to studying the *in vivo* activity and function of individual GnRH neurons. The coverage area of the 32-channel linear electrode array (a rectangular arrangement with 4 rows and 8 columns) is nearly four times larger than that of the 32-channel optic fiber electrode array (two circular layers with optics in the center). Specifically, the 32-channel linear electrodes can cover a 1.89 mm^2^ brain area (0.9 mm anterior-posterior and 2.1 mm lateral), while the 32-channel optic fiber electrodes can only cover a 0.5 mm^2^ brain area (with a maximum radius of 0.4 mm). Moreover, light penetration through brain tissue is limited, resulting in a 90% power density loss at a distance of 0.5 mm from the fiber optic probe.^30^ This limitation, combined with the relatively low yield in identifying units,^31^ makes it very challenging to detect GnRH neurons using optogenetics.

We also employed an additional identification method of kisspeptin-induced excitation to identify GnRH neurons. Peripherally administered kisspeptin induces electrophysiological changes in GnRH cells because GnRH neurons extend complex and highly branched dendritic trees beyond the blood-brain barrier (BBB) into the organum vasculum of the lamina terminalis (OVLT).^32^ Both Kiss-54 and Kiss-10 are widely used in reproductive axis studies. In agreement with others, we find that peripherally administered Kiss-54 (Figure 1B; LH increasing duration: ∼2.5 h, LH peak: ∼1 h) sustains LH release significantly longer than Kiss-10 (Figures S1B and S1C; LH increasing duration: ∼0.5 h, LH peak: ∼10 min). This prolonged effect allows sufficient time for blood sampling and reduces stress in mice. In contrast, Kiss-10 exhibits direct and repeatable dose-dependent effects.^33^^,^^34^ The transient excitatory effect of Kiss-10 on GnRH neurons (Figure S2A) provides a swift and transient response, which is advantageous for reducing the duration of recordings, making it more suitable for electrophysiological studies. Therefore, we use Kiss-54 when assessing blood LH levels due to its long half-life, while employing Kiss-10 for electrophysiological recordings of GnRH neurons based on its short half-life and dose-dependent effects, thereby minimizing the time interval between experiments. Our strategy of employing dual criteria is conservative but more stringent than relying on a single approach.

The *in vivo* findings of GnRH neurons in our research differ significantly from previous brain slice studies. Prior research has used extracellular recordings in acute brain slices to measure individual GnRH neuron activity,^35^ providing valuable insights into their intrinsic membrane properties and spontaneous firing patterns, which range from complete quiescence to irregular and tonic firing, as well as bursting *in vitro*.^36^ Our *in-vivo* mean baseline firing rate of GnRH neurons is approximately 5 Hz, which is significantly higher than the recordings obtained from *in vitro* brain slices,^35^ but somewhat more comparable to the findings from anesthetized *in vivo* studies conducted by Constantin.^20^ The studies conducted on hypothalamic magnocellular neurons have demonstrated that burst firing is more enhanced but also more variable *in vivo* compared to *in vitro* conditions.^37^ Additionally, it has been observed that the frequency of postsynaptic potentials in these cells is significantly reduced when studied *in vitro* as opposed to *in vivo*.^38^^,^^39^ These findings strongly suggest that the less variable firing patterns exhibited by GnRH neurons *in vitro* are a result of the absence of their normal synaptic inputs. Given that the population of GnRH neurons is intermingled with functionally distinct subtypes,^40^^,^^41^^,^^42^ the absence of normal synaptic inputs to GnRH neurons in acute brain slices implies a disparity between their firing patterns *in vivo* and *in vitro* conditions.^20^

Interestingly, GnRH neurons *in vivo* exhibit irregular, oscillatory patterns of activity throughout the estrous cycle. However, as anticipated, our research did not find any correlation between GnRH neuron firing rates and expected pulsatile LH secretion profiles. It is well-established that GnRH pulses and surges play a crucial role in female reproductive cyclical activity and spontaneous ovulation.^6^ Pulsatile LH secretion occurs approximately once per hour during metestrus, diestrus, and proestrus stages but is much less frequent during estrus.^8^^,^^43^ Baseline GnRH neuron activity in female mice was recorded between 10:00 a.m. and 12:00 p.m. during metestrus, diestrus, proestrus, and estrus. This time frame is commonly used to record LH pulses^43^^,^^44^ without interference from the LH surge, so we record this time window for a more accurate representation of the basal state of GnRH neurons. In our study, we found that the irregular and oscillatory patterns of *in-vivo* rPOA GnRH neuron activity continued throughout the cycle and did not mirror LH pulse secretion.^8^^,^^43^ Our investigation into the *in-vivo* basal excitatory properties of rPOA GnRH neurons demonstrates a notable increase in firing rates during proestrus when compared to estrus and metestrus, suggesting a dynamic fluctuation throughout various stages of the estrous cycle. Diestrus signifies an extended phase marked by baseline electrophysiological activity levels of GnRH neurons positioned between the "quiescent" state observed during estrus and metestrus, as well as the heightened activity displayed during proestrus.

Our research reveals that approximately half of rPOA GnRH neurons are involved in the LH surge, while the remaining half display either a "quiescent" state or rhythmic activity during proestrus and diestrus. The activity of GnRH neurons during the GnRH/LH surge was recorded over a 20-h period from 14:00 on proestrus to 10:00 the following morning. The onset of c-Fos expression in GnRH neurons during proestrus correlates strongly with increased LH secretion,^13^ which begins approximately 2 h before lights-off and persists over a 4-h window.^9^ A recent study using GCaMP technology to investigate the oscillatory activity of mouse GnRH neuron dendrites *in vivo* during proestrus demonstrated a gradual increase in baseline activity starting 4 h before lights-off and returning to baseline by 12.6 h after lights-off. Therefore, we chose to record a 20-h period from 14:00 p.m. (5 h before lights-off) to 10:00 a.m. (15 h after lights-off), effectively capturing the excitation period of GnRH neurons during the GnRH/LH surge.^45^

We show here that the “surge” GnRH neurons exhibit an increase in firing beginning early in the afternoon, several hours before “lights off” and the expected time of onset of the LH surge. In the present study, it has been surprising to observe significant variations in the firing patterns of “surge” GnRH neurons during a 20-h recording period in proestrus, with even the same neuron exhibiting diverse firing patterns across different recording periods. In our study, some GnRH neurons exhibited oscillatory and relatively consistent increases in firing rate, while others exhibited transient increments near lights-off or displayed relatively limited dynamic changes in activity during proestrus. Previous brain slice studies have reported that approximately 40% of rPOA GnRH neurons are silent, whereas 5% exhibit tonic firing and 55% display burst firing.^17^^,^^46^ Substantial evidence suggests that the soma-proximal dendritic region of rPOA GnRH neurons plays a crucial role in generating LH surges.^4^^,^^47^ Our study implies that the activity of GnRH neurons is not static and that the occurrence of the LH surges may result from a cumulative effect involving the variable activity of all LH surge-related GnRH neurons.

### Limitations of the study

It is important to note that the observation that approximately half of rPOA GnRH neurons are involved in LH surge should be considered as an approximate reference value due to limitations from the false-negative detection of GnRH neuron numbers and potential influence from stress on the population involved during our recordings. Using a 32-nickel-chromium electrode wire array to maximize anatomical coverage of rPOA, we found that around 15% (15 out of 97 recorded units in 6 mice) could be identified as GnRH neurons, which is quite similar to Constantin’s study.^20^ The nature of the 32-channel fixed electrodes we used means that the recorded signals could not achieve pure single units, which can only be achieved by tetrode and Neuropixel recordings. However, although Tetrode technology ensures the accuracy of single-neuron firing, it is only suitable for brain regions where neuronal cell bodies are densely arranged, such as the CA1 pyramidal neurons, which form a compact layer consisting of 5–8 superimposed rows of pyramidalneurons.^48^^,^^49^ In contrast to most central nervous system neurons, the GnRH neuron cell bodies are widely scattered within the basal forebrain. In our preliminary experiments using tetrode technology, no effective recordings of GnRH neurons were obtained using this method. The coverage area of a single electrode with 32 fixed channels is significantly larger than that of tetrodes, which increases the likelihood of capturing GnRH neurons located deep within the brain and scattered throughout this region.^3^ We use dual identification criteria with kiss and CNO to identify GnRH neurons more accurately, but we cannot reach single-unit isolation at present. Although *in vivo* electrophysiological recording combined with pharmacological and chemogenetic approaches is currently an appropriate method for identifying GnRH neurons, direct evidence for their definitive identification remains an area of ongoing investigation.

In summary, we present the initial direct recordings of rPOA GnRH neuron activity in freely moving female mice using chemogenetic and *in vivo* electrophysiology techniques. Firstly, we employed GnRH::DIO-hM4D(Gi) and GnRH::DIO-hM3D(Gq) female mice injected with Kiss10 and/or CNO to double identify GnRH neurons. Secondly, we recorded the basic electrical activity of rPOA GnRH neurons throughout different estrous cycles and observed a significant increase in firing rates during proestrus compared to metestrus and estrus. Subsequently, we clearly identified one subtype of GnRH neurons exhibiting LH surge-like excitability during proestrus while maintaining quiescence during diestrus. Interestingly, the firing patterns of this specific subtype of GnRH neurons exhibit significant variations at the time of LH surge: some neurons display prolonged heightened activity near lights-off, while others demonstrate brief bursts, and certain neurons exhibit distinct oscillatory activity. Furthermore, even within the same GnRH neuron, the firing patterns differ during LH surges. Another subtype remained "quiescent" or displayed rhythmic activity throughout both proestrus and diestrus. This study represents a significant milestone in the functional classification of GnRH neurons and holds great importance for research on female reproductive neuroendocrine regulation.

## Resource availability

### Lead contact

Further information and requests for resources and reagents should be directed to and will be fulfilled by the lead contact, Wang Li (wanglishfd@126.com).

### Materials availability

This study did not generate new unique reagents.

### Data and code availability

All data reported in this article will be shared by the lead contact upon request. This article does not report original code. Any additional information required to reanalyze the data reported in this article is available from the lead contact upon request.

## Acknowledgments

The study was financially supported by the 10.13039/501100001809National Natural Science Foundation of China (82271732 to YP. K, 82471738 to L.W, 82001502 to Y.L., 82201888 to X.S.). The authors thank all members of the lab for their support.

## Author contributions

Wang Li, Allan E. Herbison, and Kuang Yanping conceived and designed the experiments. Liu Yali and Shen Xi performed surgical procedures on mice. Zeng Yuqi and Nie Yunhan conducted the LH test and Immunofluorescence analysis. Liu Yali and Xu Jiamin analyzed all the data obtained from the experiments. Wang Li and Liu Yali prepared for the figures. The article was written by Wang Li and Liu Yali with contributions from all authors.

## Declaration of interests

The authors report no competing interests.

## Declaration of generative AI and AI-assisted technologies

The authors declare that no generative AI or AI-assisted tools were used to create or alter images in submitted articles.

## STAR★Methods

### Key resources table

**Table:** 

REAGENT or RESOURCE	SOURCE	IDENTIFIER
**Antibodies**		

Polyclonal rabbit anti-GnRH antisera	Gift G. Anderson, University of Otago	Cat #GA01 RRID:AB_2721114
Cy3-AffiniPure Donkey Anti-Rabbit IgG (H+L)	Jackson Immuno Research Labs	Cat# 711-165-152, RRID:AB_2307443
Monoclonal anti-bovine LH 518B7	University of California	RRID:AB_2665514
Anti-LH AFP 240580	National Hormone & Peptide Program, Torrance, California)	RRID:AB_2665533
Goat Anti-Rabbit IgG H&L (HRP)	Abcam	RRID:AB_955447

**Bacterial and virus strains**		

AAV2/9 hSyn-DIO-hM4D(Gi)-EYFP-WPRE-hGH.polyA	BrainVTA(Wuhan) Co., Ltd	PT-0344 Viral titer:5.54 × 10ˆ12 vg/mL
AAV2/9 EF1a-DIO-hM3D(Gq)-EYFP-WPRE-hGH.polyA	BrainVTA(Wuhan) Co., Ltd	PT-0816 Viral titer:5.33 × 10ˆ12 vg/mL

**Chemicals, peptides, and recombinant proteins**		

Kisspeptin-10	Tocris	Cat# 2570
Kisspeptin-54	Tocris	Cat# 1443
clozapine-N- oxide	Sigma Aldrich	P0130
Mouse LH standard	NIDDK-NHPP	AFP-5306A
Dexamethasone	MedChemExpress	Cat# HY-14648

**Experimental models: Organisms/strains**		

STOCK Tg(Gnrh1-cre)1Dlc/J	The Jackson Laboratory	Strain #:021207 RRID:IMSR_JAX:021207

**Software and algorithms**		

GraphPad Prism software	GraphPad Prism	RRID:SCR_002798
ImageJ image analysis software	ImageJ	RRID:SCR_003070
Plexon MAP system	Plexon, Dallas, TX, USA	RRID:SCR_003170
Neuroexplore	Plexon, Dallas, TX, USA	RRID:SCR_001818
Offline Sorter	Plexon, Dallas, TX, USA	RRID:SCR_000012

**Other**		

Olympus confocal microscope	Olympus	FV3000
Cryomicrotome	Leica Biosystems, United States	CM1860
Microscope cover glass	CITOTEST	Cat# 10212450C
Custom 32-channel electrode	Nanjing Greathink Medical Technology Co., Ltd	btame04l

### Experimental model and study participant details

#### Animals

The STOCK Tg(Gnrh1-cre)1Dlc/J mice, in which the Cre recombinase is targeted to the first coding exon of Gnrh1^47^ (JAX #021207), were crossed with Gnrh1-Cre^+/-^ mice. Genotypes were determined by PCR analysis using mouse tail DNA samples. Gnrh1-Cre^+/-^ and WT female mice aged 8–12 weeks were used for this experiment when monitoring two regular estrous cycles. All mice were housed under a 12:12 hr lighting schedule (lights on at 07:00-19:00) and provided *ad libitum* access to food and water enriched with nesting material. All experiments were conducted in accordance with the guidelines approved by the Animal Care and Use Committee of Shanghai Jiao Tong University School of Medicine (SH9H-2023-A869-1).

### Method details

#### Stereotaxic administration of AAVs into the rPOA region of Gnrh1-Cre^+/-^ mice

For the chemogenetic inhibition and activation studies, recombinant cre-dependent AAV2/9 hSyn-DIO-hM4D(Gi)-EYFP-WPRE-hGH.polyA, or AAV2/9 EF1a-DIO-hM3D(Gq)-EYFP-WPRE-hGH.polyA (≥5 × 10^12^ GC/mL; BrainVTA (Wuhan) Co., Ltd) were injected bilaterally into the region of the rPOA of Gnrh1-Cre^+/-^ mice. Adult female mice were anesthetized with 2% isoflurane and placed in a stereotaxic apparatus. The skull was exposed through a small incision and holes were drilled for virus injection. Bilateral injections were performed using a Hamilton syringe apparatus equipped with a 10 μL Hamilton syringe with 25-gauge needle. The coordinates for viral delivery into the rPOA were obtained from mouse brain atlas of Paxinos and Franklin^50^: AP 0.6 mm; ML ±0.3 mm; DV -4.8/-5.0 mm. The needle was carefully lowered into place at each of the four injection sites, injecting 0.1μL AAV over a period of five minutes per site, and leaving the needle *in situ* for an additional five minutes before withdrawal.

#### Tail-tip blood sampling and LH ELISA

Two to 3 weeks after surgery, freely behaving AAV-injected Gnrh1-cre mice were given Kiss54 (1 nmol, Intraperitoneal injection, Tocris) or CNO ((2 mg/kg, Intraperitoneal injection, Sigma Aldrich) and repetitive tail-tip pulse blood sampling undertaken. Following a 1 h acclimation period, blood samples (6μl) were collected using the tail-tip method.^51^ For GnRH-Cre:: hM4D(Gi) mice, blood samples were taken 30 min before, at the time of Kiss-54 injection, and then 10 and 20 min later followed by the injection of CNO at 30 min and subsequent blood samples every 30mins for 120min. For GnRH-Cre::hM3D(Gq) mice, blood samples were taken 30 min before, at the time of CNO injection and then 10, 20, 30, 60, 90 min later. Whole blood (6ul) was immediately diluted in 114μL of 0.1M PBS with 0.05% Tween 20, vortexed, and snap frozen on dry ice, then stored at -20°C for a subsequent LH ELISA.^51^ The LH concentration was quantified utilizing a mouse LH standard (mLH; reference preparation, AFP-5306A, NIDDK-NHPP, USA), a coating antibody (monoclonal anti-bovine LH beta subunit antiserum, 518B7, University of California, CA, USA), an anti-LH antibody (anti-LH AFP 240580, National Hormone & Peptide Program, CA, USA), and Goat Anti-Rabbit IgG H&L (HRP) (Abcam, Cambridge, United Kingdom) as the secondary antibody. The concentration of LH in whole blood samples was determined by interpolating the optical density values against a nonlinear regression of the LH standard curve. The assay sensitivity was 0.04 ng/mL, with an intra-assay coefficient of variation of 7.2%.^52^

Those GnRH-Cre::hM4D(Gi) mice exhibiting an increase in LH levels following the administration of Kiss-54 and a decrease after subsequent reinjection of Kiss-54+CNO were selected for the implantation of the 32 fixed channel electrode array. Similarly, those GnRH-Cre::hM3D(Gq) mice with an increase in LH levels following the injection of CNO were subsequently electrode implantation.

#### Implantation of 32-fixed channel electrodes into the rPOA region of Gnrh1-Cre^+/-^ mice

The mice were anesthetized using 2% isoflurane and mounted in a stereotactic frame. To maintain a constant body temperature, a small-animal thermoregulation device was utilized. Dexamethasone (10 mg/kg, s.c, MedChemExpress) was administered to prevent cranial swelling. Markers were placed at positions 1.2mm and 0.2mm before Bregma, as well as positions 1.2mm on both sides, indicating the boundaries surrounding the fixed channel electrode array (Nanjing Greathink Medical Technology Co., Ltd). This electrode array consists of 32 nickel-chromium wires arranged in four rows and eight columns with a distance of 300um between each wire (Figure 1I). The anatomical coverage of the electrode extends 0.9 mm anteriorly and posteriorly, as well as 2.1 mm laterally providing comprehensive rPOA coverage.

The rectangular section of the skull was surgically removed based on the coordinates of the aforementioned four points. The outermost electrode was inserted to a depth of 5.4 mm, while the remaining three rows of electrodes were positioned at depths of 5.1 mm, 4.9 mm, and 4.6 mm from outside to inside. Two small holes were drilled in the dorsal plates of the mouse skull and the fixation screws were inserted into these respective holes. One of the screws was soldered with copper wire as a grounding reference. Any gaps between the electrodes and craniotomy were filled with softened paraffin before securing 32 fixed electrodes onto the skull using dental cement and screws. Following surgery, individual animals were housed in round open-top cages (35 cm diameter, 45 cm height) under a 12-hour light/dark cycle with *ad libitum* access to food and water for a recovery period of two weeks.

#### The in-vivo monitoring of neuronal spiking activity in freely behaving mice

The microelectrode connectors were connected to preamplifiers with extended cables in order to monitor neuronal signals using the Plexon MAP system (Plexon, Dallas, TX, USA). The recorded spike waveforms and time stamps were saved as Plexon data (∗.plx) files. We utilized Offline Sorter software (Plexon, Dallas, TX, USA), which is provided with the multi-channel recording system, for preprocessing raw neuronal discharge data. The extracellular signals from electrodes were filtered through preamplifiers and a 300 Hz high-pass fliter to isolate spiking activity.^53^

Extracellular signals from electrodes were filtered using preamplifiers and a 300 Hz high-pass filter to isolate spiking activity.^53^ Action potentials were identified as rapid events (∼1 ms duration) with amplitudes exceeding twice the background noise level.^21^ After detecting putative spike events via an amplitude threshold of 64 μV, these events were time-aligned and projected into a lower-dimensional space. Principal component analysis (PCA) was employed to distinguish the discharge patterns of individual neurons.^54^^,^^55^ We combined the first principal components with various wavelet coefficients, including spike amplitude, width, and energy, to capture most of the information contained in the original variables.^56^ Following clustering of neuronal activities in the feature space, we eliminated clusters reflecting random variations by grouping waveforms with similar shapes and removing invalid waveforms.^56^^,^^57^ The data processed by Offline Sorter was subsequently imported into Neuroexplore software (Plexon, Dallas, TX, USA) for further data analysis.

#### The identification of GnRH neurons *in vivo*

The GnRH neurons in GnRH::hM4D(Gi) mice were identified through the administration of Kiss-10(10 nmol, Intraperitoneal injection, Tocris) and Kiss10+CNO with the activation of firing by Kiss-10 needing to be reduced by >40% by CNO.^58^^,^^59^^,^^60^^,^^61^^,^^62^^,^^63^ The excitatory rate induced by Kiss-10 was calculated from the change in firing rate during the first 10 minutes after Kiss-10 injection and baseline firing rates over a 30-minute period. Similarly, the excitatory rate induced by both Kiss-10 and CNO was determined from the change in firing rate during a 30-minute period following co-injection of Kiss-10 with CNO compared to baseline firing in the preceeding 30-minute period. The GnRH neurons in GnRH:hM3D mice were identified through the administration of Kiss10 and CNO that both needed to increase firing by >40%.^58^^,^^59^^,^^60^^,^^61^^,^^62^^,^^63^ The excitatory rate induced by CNO was calculated from the firing rate during the first 30 minutes after CNO injection and the baseline firing rates 30 min prior.

#### Recording the in-vivo electrophysiological activity of GnRH neurons

The estrous cycles of female GnRH::hM4D(Gi) and GnRH::hM3D(Gq) mice were characterized by daily vaginal smearing over at least 2 weeks before starting the experiment and only regularly cycling mice used. Estrous cycles stage^64^ was evaluated by vaginal smears collected at 8:00 - 9:00 h. The basal activities of GnRH neurons in freely behaving mice were recorded for 2-hour periods between 10 am and 12 pm during different estrous cycles. Only GnRH neurons that met the aforementioned dual identification criteria were eligible for further data analysis. The firing rates, with bins set at intervals of every 5 and 300 seconds, were analyzed for metestrus, diestrus, proestrus, and estrus.

For extended 20-h recordings across proestrus into estrus, the microelectrode was connected to the recording system from 14:00 until 10:00 on the following day. A helium-filled mylar balloon was attached to the cables to reduce their weight and enable unrestricted movement of the mouse. Mice were provided with the normal diet and water and their activity recorded throughout the 20h period after which the microelectrode array was disconnected. Subsequent recordings were conducted 3-5 days later depending on the estrous cycle stage by repeating the above procedure.

#### Immunofluorescence

Mice were anesthetized with 2% isoflurane and perfused with saline, followed by 4% paraformaldehyde in 0.1M PBS. The brains were promptly removed, post-fixed overnight in 4% paraformaldehyde, and subjected to dehydration using increasing saccharin solutions (20–30%) at 4°C. Frozen brain slices were sectioned at a thickness of 25 mm on a cryomicrotome (Leica CM1860, Leica Biosystems, United States). Coronal brain sections taken throughout the rPOA were incubated in a solution containing 3% normal horse serum and 0.3% Triton-X for one hour, followed by overnight incubation with rabbit anti-GnRH antisera (GA3, 1:2000 dilution; gift from Greg Anderson, University of Otago) at 4°C. After rinsing the slices in PBS, they were further incubated with donkey anti-rabbit Cy3 antibody (1:1000 dilution; Jackson Immunoresearch). Dual fluorescence images were captured using an Olympus FV3000 confocal microscope.

### Quantification and statistical analysis

Statistical analyses were conducted using GraphPad Prism software (GraphPad Software). Two-way repeated measures ANOVA with Holm-Sidak test were employed for comparing LH levels within two groups, as well as the firing rates during the proestrus phase and those during diestrus. Parametric one-way ANOVA, followed by post hoc Tukey’s multiple comparisons test, was used to compare the basal firing rates of GnRH neurons across different estrus cycles. Statistical significance was considered at p < 0.05. The values are presented as mean ± SEM.

### Additional resources

The animal procedures were conducted in accordance with the guidelines set by the institutional animal care committee of Shanghai Ninth People’s Hospital (SH9H-2023-A869-1).

## Supplemental information

Document S1. Figures S1–S4

Data S1. Review of iScience article
