# Supplementary material for: In-vivo properties and functional subtypes of gonadotropin-releasing hormone neurons
Source: iScience. 2025 Apr 22;28(5):112513. doi: 10.1016/j.isci.2025.112513 (PMC12124600; doi:10.1016/j.isci.2025.112513)
Supplement: Document S1. Figures S1–S4 [file mmc1.pdf]

**iScience, Volume *28***

## Supplemental information

# In-vivo properties and functional subtypes

# of gonadotropin-releasing hormone neurons

**Yali Liu, Xi Shen, Yuqi Zeng, Yunhan Nie, Jiamin Xu, Allan E. Herbison, Yanping Kuang, and Li Wang**

## Figure S1

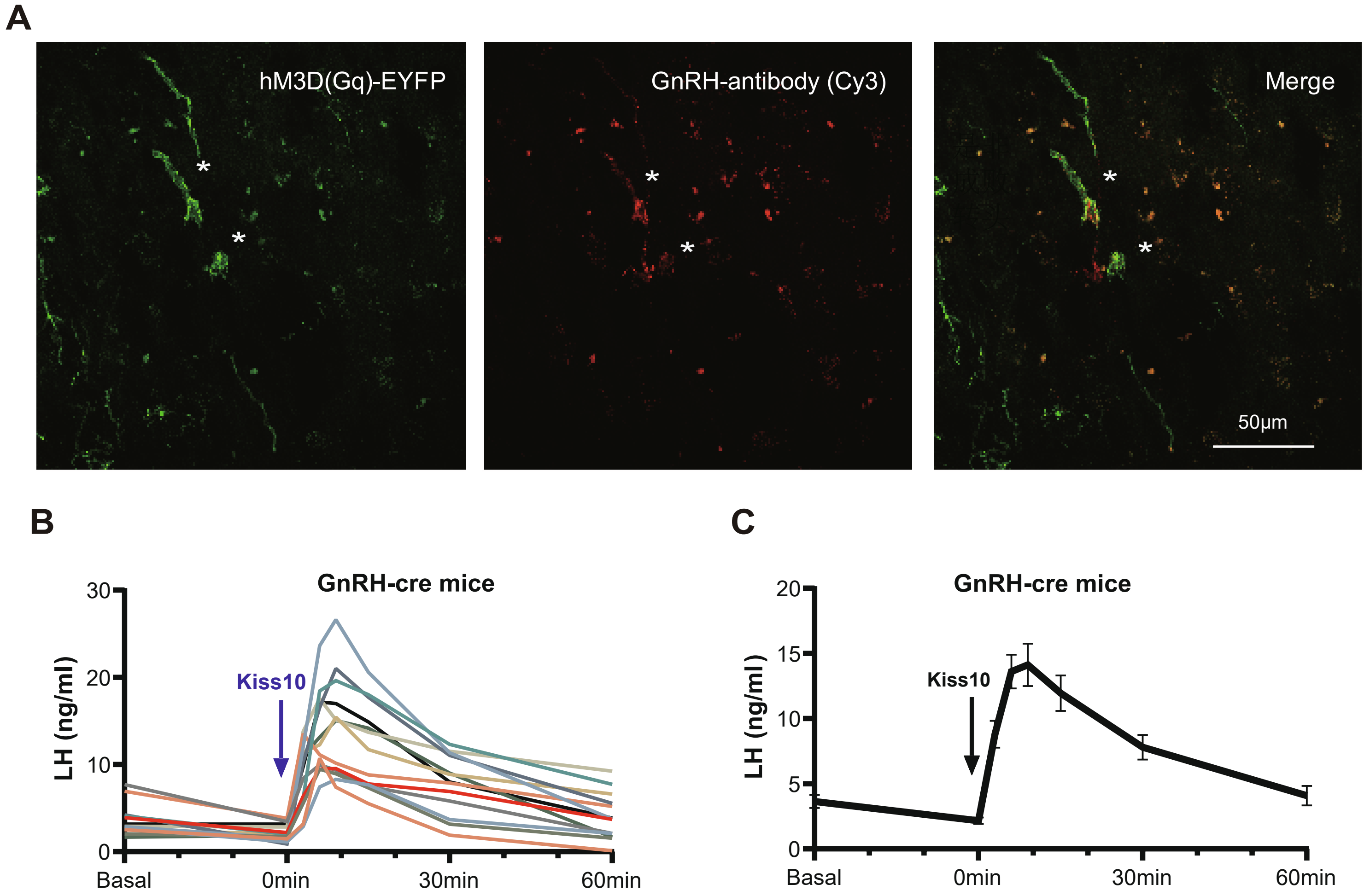

A
hM3D(Gq)-EYFP
GnRH-antibody (Cy3)
Merge
50µm
B
GnRH-cre mice
Kiss10
LH (ng/ml)
30
20
10
0
Basal
0min
30min
60min
C
GnRH-cre mice
Kiss10
LH (ng/ml)
20
15
10
5
0
Basal
0min
30min
60min

## Figure S2

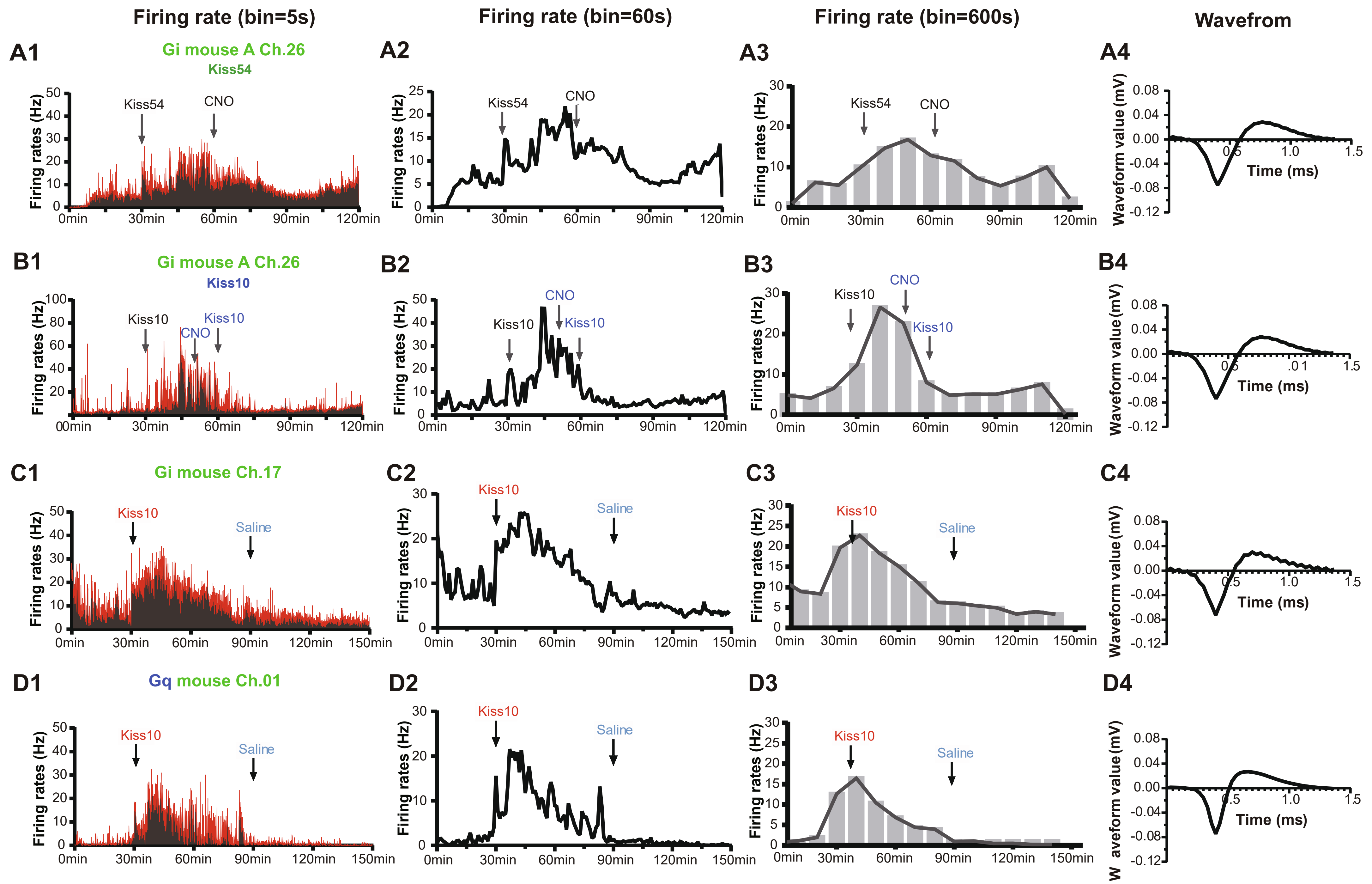

Firing rate (bin=5s)
Firing rate (bin=60s)
Firing rate (bin=600s)
Wavefrom
A1
Gi mouse A Ch.26
Kiss54
CNO
Firing rates (Hz)
0min
30min
60min
90min
120min
A2
A3
A4
Waveform value (mV)
Time (ms)
B1
Gi mouse A Ch.26
Kiss10
CNO
00min
B2
B3
B4
C1
Gi mouse Ch.17
Saline
150min
C2
C3
C4
D1
Gq mouse Ch.01
D2
D3
D4

Figure S3

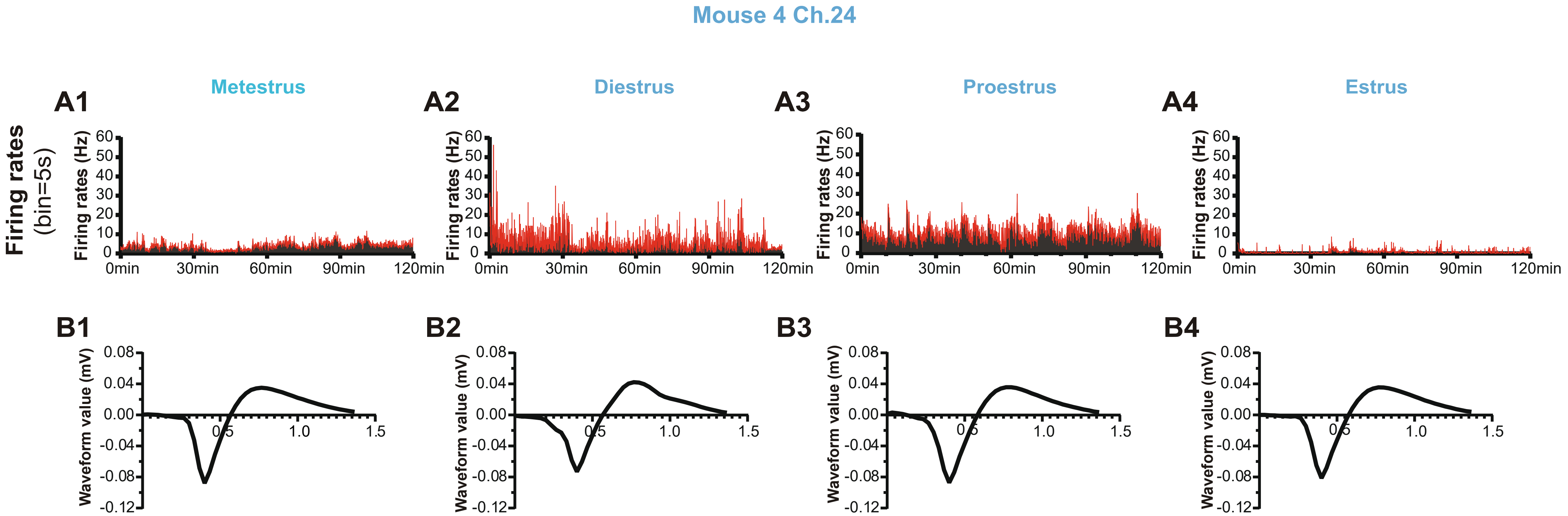
Mouse 4 Ch.24
A1
Metestrus
A2
Diestrus
A3
Proestrus
A4
Estrus
Firing rates
(bin=5s)
Firing rates (Hz)
0
10
20
30
40
50
60
0min
30min
60min
90min
120min
B1
B2
B3
B4
Waveform value (mV)
0.08
0.04
0.00
-0.04
-0.08
-0.12
0.5
1.0
1.5

## Figure S4

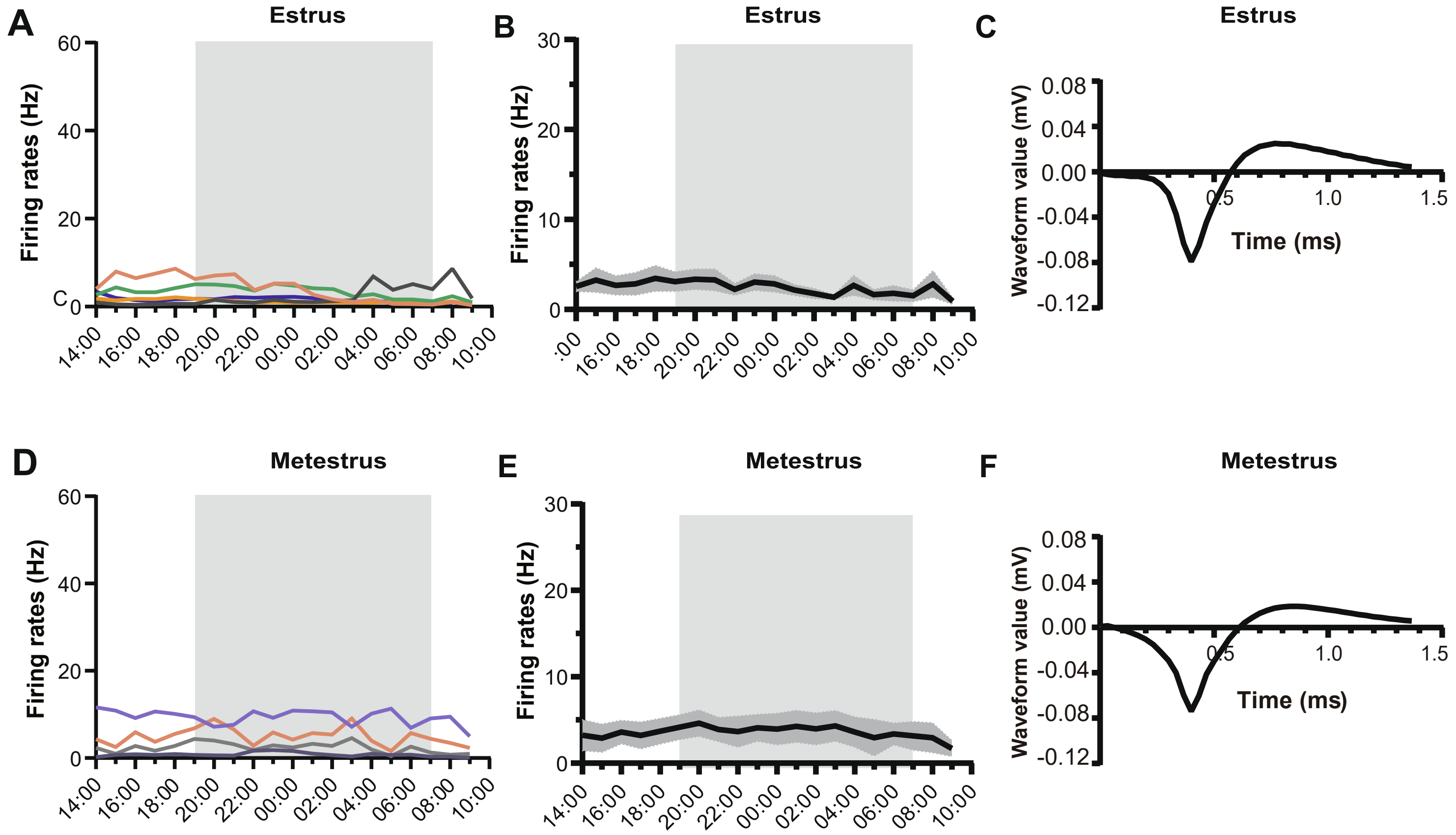

A
Estrus
Firing rates (Hz)
60
40
20
0
14:00
16:00
18:00
20:00
22:00
00:00
02:00
04:00
06:00
08:00
10:00
B
Estrus
Firing rates (Hz)
30
20
10
0
C
Estrus
Waveform value (mV)
0.08
0.04
0.00
-0.04
-0.08
-0.12
0.5
1.0
1.5
Time (ms)
D
Metestrus
Firing rates (Hz)
E
Metestrus
Firing rates (Hz)
F
Metestrus
Waveform value (mV)
Time (ms)

**Figure S1. Peripheral administration of Kiss10 stimulates plasma LH levels in female Gnrh1-Cre$^{+/-}$ mice, related to Figure 1.**

(A) The Photomicrographs depict the presence of GnRH immunoreactivity (in red), hM3D(Gq)- EYFP expression (in green), and their overlay in GnRH-Cre mice that received bilateral injections of Cre-dependent DIO-hM3D(Gq)-EYFP AAVs into the rostral preoptic area (rPOA). Asterisks indicate GnRH neurons expressing hM3D(Gq)-EYFP. Scale bar, 50 µm. (B-C) The LH profiles of Gnrh1-Cre$^{+/-}$ mice administered with Kiss10 alone (n = 13) were obtained individually and merged, and the results were presented as mean ± SEM.

**Figure S2. Examples of the same GnRH neuron identified in the GnRH::DIO-hM4D(Gi) mice through both Kiss 54 and kiss 10, following by CNO injection, related to Figure 2.**

The firing rates of the same GnRH neuron were stimulated by Kiss54 with CNO (A1-A3) and subsequently by Kiss10 in combination with CNO+kiss10 again (B1-B3). The waveforms of the same GnRH neuron showed no difference when stimulated by either Kiss54 (A4) or Kiss10 (B4). Saline-injected control groups in both the GnRH::hM4D(Gi) and GnRH::hM3D(Gq) mouse lines (C1-D4).

**Figure S3. The Profiles of basal firing rates in the morning of the same GnRH neuron across four different estrous, related to Figure 3.**

(A1-B4) The basal firing rates (bin=5s) of the same GnRH neuron across different stages of the estrous cycle, recorded from 10:00-12:00AM (A1-A4), along with their corresponding waveforms (B1-B4).

**Figure S4. In-vivo electrophysiological activities of GnRH neurons recorded overnight during estrus and metestrus, related to Figure 4-6.**

(A) Firing rates (bin=3600s) of GnRH neurons across the transition from estrus to metestrus (n=5 recordings from 3 neurons in 2 mice), along with the mean (±SEM) firing rate levels (B) and mean (±SEM) waveform (C). (D) Firing rates (bin=3600s) of GnRH neurons across the transition from metestrus to diestrus (n=5 recordings from 4 neurons in 2 mice), along with the mean (±SEM) firing rate levels (E) and mean (±SEM) waveform (F).
